# The Cost of Inpatient Care of Schizophrenia and Treatment Schedules Used in German Academic Center: Kiel

**DOI:** 10.1007/s11126-015-9412-0

**Published:** 2015-12-21

**Authors:** Tomasz Zaprutko, Robert Göder, Krzysztof Kus, Lyudmyla Rakhman, Rostyslav Bilobryvka, Elżbieta Nowakowska

**Affiliations:** 1 Department of Pharmacoeconomics and Social Pharmacy, Poznan University of Medical Sciences, 79 Dąbrowskiego Street, 60529 Poznan, Poland; 2 Department of Psychiatry and Psychotherapy, Christian-Albrechts-Universität zu Kiel, 147 Niemannsweg Street, 24105 Kiel, Germany; 3 Department of Psychiatry, Psychology and Sexology, Lviv National Medical University, 95 Kulparkivska Street, L’viv, 79021 Ukraine

**Keywords:** Schizophrenia, Inpatient care, Direct costs, Therapy

## Abstract

The authors aimed at analyzing the costs of inpatient care of schizophrenia in Kiel (Germany). The study was also to present treatment regimens used at the German Academic Center. Moreover, the study is a continuation and complement of the previous study conducted in Polish and Ukrainian Academic Center. Therefore, it helps increase the awareness and knowledge of residents concerning the cost of inpatient care of schizophrenia. The analysis was based on 105 hospital records of patients treated between January 2012 and June 2013. According to inclusion criteria, 50 adult patients (27 women and 23 men) were included in the study. The study was approved by the Ethics Committee of the Medicine Faculty of CAU in Kiel. The cost of schizophrenia treatment of 50 patients in Kiel was EUR 604,280.90 ($\bar{x}$ = EUR 12,085.62). The duration of hospital stay was on average $\bar{x}$ = 51.02 days. The patients were treated with neuroleptics of all generations. The most popular atypical neuroleptic was amisulpride and the most popular typical neuroleptic was haloperidol. Patients from Kiel were provided a comprehensive non-pharmacological treatment. Treatment regiments and evaluations of costs of schizophrenia vary between countries. The costs of inpatient care of schizophrenia are high in Kiel. Treatment of schizophrenia seems to be comprehensive in Kiel and wide range of treatment opportunities contribute to a more effective treatment confirmed by less frequent relapses of schizophrenia than in Lviv (Ukraine), for example. Comprehensive treatment should be available everywhere, because it is a right of every patient.

## Introduction

Schizophrenia is considered one of the most disabling and persistent mental disorder [1] characterized by severe and recurring course with a number of peculiar symptoms [2]. It requires long-lasting and comprehensive treatment [1, 3]. Moreover, schizophrenia causes functional disruption in many dimensions like interpersonal relationships, self-care and work [4].

The lifetime prevalence of schizophrenia is approximately 1 % [2] and the disorder is frequently associated with stigmatization and social rejection [4]. It has a significant health and economic burden [1] with wide-ranging impacts not only for the patients themselves but also for their families and for the society [5]. Several reasons of this situation have been proposed [6] but the majority of them are related to direct and indirect costs which are significant in schizophrenia [4] and contribute to the statement that schizophrenia has been identified as the most expensive disease among all psychiatric disorders in terms of health care outlay per patient [7, 8].

Although estimations concerning costs of schizophrenia vary between countries, two main categories of costs are identified in research studies [9]. Direct costs include mainly expenses for inpatient and outpatient care, and medications [5, 6]. Indirect costs, however, usually arise from the lost productivity of patients and their healthy caregivers [5, 6]. In the UK and American studies, the estimated total cost of schizophrenia was £6.7 billion and US $62.7 billion, respectively, with indirect costs accounting for about 2/3 of total costs [5, 10]. Despite this, calculations concerning direct costs of schizophrenia are as significant as studies on indirect costs because comprehensive care during inpatient care is frequently the basis for effective treatment in the long run. Furthermore, the scale of costs observed in both categories confirms the need of conducting several analyses, which present aspects related to economy of mental health.

This paper focuses on the economic burden of schizophrenia treatment in the Department of Psychiatry and Psychotherapy of the Christian-Albrecht University (CAU) Hospital in Kiel (Germany) and treatment regimens used there. Moreover, the analysis is a development and continuation of the previous study conducted in Poznan (Poland) and Lviv (Ukraine) [11] and contributes to the interesting comparison of results obtained in three different though nearby countries. Our study will be useful both for health care decision-makers, residents and for clinicians as well. The estimation of the costs of schizophrenia in the German academic center will be an important and precious contribution to the debate concerning cost-effective treatment of schizophrenia in the long run.

## Materials and Methods

The study presents the results of analysis of inpatient care costs in adult patients hospitalized at the Department of Psychiatry and Psychotherapy of Christian-Albrechts University (CAU) in Kiel (Germany). The inclusion criteria were as follows: adult age of patients of both sexes, schizophrenia diagnosed on the basis of the International Classification of Diseases, Tenth Revision (ICD-10); hospitalization started and ended between January 2012 and June 2013. Patients were excluded from the study in case of different time period of hospitalization, incomplete hospital records, hospitalizations shorter than 14 days. In the study, 105 hospital records were analyzed and, according to the study criteria, 50 (27 women and 23 men) hospital records were included. Necessary permissions to conduct analysis were obtained from hospital decision-makers in Kiel and from the Ethics Committee of the Medicine Faculty of CAU in Kiel. What is also important, the study conforms with the Act on Protection of Personal Data. Sample can be treated as representative, because the analyzed records concerned a wide range of adult patients from a crucial medical and social center where patients are admitted from the entire region.

Information concerning patient metrics, duration of hospitalization, pharmacotherapy and non-pharmacological treatment, diagnostic tests, and relapses was gathered from hospital records. Data on prices and money value of hospital procedures were obtained from the hospital accounting departments. The cost of inpatient care of schizophrenia in Kiel is presented as a total cost, including hospital stay, pharmacotherapy, non-pharmacological care and diagnostic tests. This results from the health care system in which individual components under the value of procedure per day priced at Euro (EUR) 236.88 are covered regardless of the sex of the patient. Cost of inpatient care was calculated by multiplying the duration of the patient’s hospital stay by the cost of procedure per day. Importantly, monetary values presented in the study are calculated amounts rounded according to mathematic rules. Medicine prices used in the study were derived from the hospital drugstore price list and were used to compare economic availability of pharmacotherapy to patients treated in Kiel. Furthermore, the appraisal of the frequency of schizophrenia relapses within the last 10 years was carried out in Kiel likewise. The number of inpatient stays was divided into 3 ranges: from 0 to 3 hospitalizations, from 4 to 7 stays and above 7 hospital stays.

## Statistics

The data are shown as mean values ± SEM. The data distribution pattern was not normal (unlike Gaussian function). Statistical analyses for age in years and hospitalization were conducted using the non-parametric Kruskal–Wallis test for unpaired data.

## Results

The group of 50 German patients hospitalized for a total number of 2551 days generated the total cost of inpatient care in Kiel at the value of EUR 604,280.90 ($\bar{x} =$ EUR 12,085.62). Proportionally to hospital stay, the cost of hospitalization in Kiel was EUR 343,239.12 ($\bar{x} =$ EUR 12,712.56) for women and EUR 261,041.78 ($\bar{x} =$ EUR 11,349.64) for men, respectively. All components of the total cost (cost of hospital stay, pharmacotherapy, non-pharmacological treatment, diagnostic tests) were covered by the value of medical procedure EUR 236.88 per day (Table 1).

**Table 1:** Structure of the study group

	Total	Woman	Men
	$\bar{x}$ ± SEM	$\bar{x}$ ± SEM	$\bar{x}$ ± SEM
Number of subjects	50	27	23
Mean age in years	43.88 ± 2.10 (M:44÷U/L Q:32/54)	45.81 ± 2.90 (M:45÷U/L Q:33/56)	41.61 ± 3.03^x^ (M:41÷U/L Q:31/49) NS (p = 0.3231)
Average duration of hospitalization in days	51.02 ± 4.68 (M:35.5÷U/L Q:29/66)	53.67 ± 6.82 (M:37÷U/L Q:33/66)	47.91 ± 6.39^x^ (M:36÷U/L Q:24/67) NS (p = 0.5453)
Costs of inpatient care in Kiel	12,085.62 ± 1109.61 (M:8646.12÷U/L Q:15,634.08/6869.52)	12,712.56 ± 1616.50 (M:8764.6÷U/L Q:15,634.03/7817.04)	11,349.64 ± 1513.50^x^ (M:8527.70÷U/L Q:15,870.50/5685.12) NS (p = 0.5459)
Person-days used	2551	1449	1102

*U/L Q* upper and lower quartile, *M* median, *NS* non significant, *SEM* standard error of the mean

^X^Statistically significant difference: women versus men for p < 0.05

In the analyzed group of patients from Kiel, the mean age was 43.88 years. Women (W) (45.81 years) were more than 4 years older than men (M) (41.61 years). The average length of hospitalization was $\bar{x}$ = 51.02 days. Women were hospitalized almost 6 days longer on average (53.67 days) than men (47.91 days), using 1449 and 1102 hospital days, respectively. In relation to women included into the study, the shortest and the longest hospitalization lasted 18 and 151 days. Among men, on the other hand, it was 16 and 127 days, respectively.

Of the group of patients included in the study, 4 were treated with monotherapy. 1 patient was treated only with flupentixol, 2 patients with clozapine, and 1 patient with olanzapine. Nevertheless, 46 patients received polytherapy and 38 of them were treated with 2–5 drugs, and 8 patients with 6–10 drugs. No-one was treated with more than 10 medicines.

Patients were treated with antipsychotics of all generations. The most popular medicine was amisulpride, administered to 34 % of patients. Of atypical neuroleptics, olanzapine, aripiprazole, clozapine, and risperidone were administered to 26, 10, 22 and 30 % of patients, respectively. Nevertheless, long-acting risperidone was administered to 6 % of patients from Kiel. Apart from that, quetiapine was administered to 10 % of patients and ziprasidone to 2 % of patients hospitalized in Kiel.

In the group of typical neuroleptics, the most popular was haloperidol, administered to 18 % of patients. Chlorprotixen, flupentixol, levomepromazine were administered to 16, 8, and 8 % of patients, respectively. In addition to this, the patients were treated with melperone, perazine and pipamperon which were administered to 4 % of patients.

Furthermore, adjunctive pharmacotherapy is common in schizophrenia treatment and was administered also in Kiel.

Antidepressants used included bupropion, citalopram, escitalopram, fluoxetine, and sertraline. Mood stabilizers, anticonvulsants (sodium valproate and pregabaline), anxiolytics (buspirone), and anticholinergic agents (biperiden) were administered, too. A few patients from Kiel were treated with benzodiazepines. Apart from that, patients were treated with medicines related to concomitant disorders if necessary.

Notably, comprehensive and complex non-pharmacological treatment was provided to all patients hospitalized in Kiel. Patients participated in obligatory sessions twice a week and a physician decided whether it was psychological education, social competence training, cognitive behavioral training, or art therapy. There were also daily co-educational wards, where patients were able to spend time together and to live like in a normal community. The cost of non-pharmacological therapy was included in the permanent value of medical procedure per day.

Furthermore, assessment of frequency of schizophrenia relapses and number of hospitalizations in Kiel provides interesting data allowing evaluation of efficacy of treatment used. 0-3 inpatient stays were the case for 34 patients, whereas range from 4 to 7 hospitalizations applied to 13 patients. In a group above seven hospital stays there were 3 patients.

## Discussion

The evaluated costs of inpatient care of 50 patients affected with schizophrenia and treated in Kiel proved to be high (EUR 604,280.90; $\bar{x} =$ EUR 12,085.62). This amount is higher than in Poznań (Poland)—EUR 160,572.08 (n = 50; $\bar{x} =$ EUR 3,211.44) or Lviv (Ukraine)—EUR 30,943.37 (n = 58; $\bar{x} =$ EUR 533.50) [11]. The degree of the discrepancy observed is not surprising, however, but common, with difference presented by Salize et al. [12] who showed a 12-fold difference in costs of schizophrenia treatment between hospitals from Switzerland and Spain and a sevenfold difference between hospitals from Switzerland and England. Similar discrepancies were observed in studies conducted by Andlin-Sobocki et al. [13] and by Zhai et al. [14] who presented a sixfold difference between Estonia and Switzerland and sevenfold between China and Switzerland, respectively. It is also crucial that international and comparative analyses are difficult to conduct and to present, likewise, because not only different health care systems but also different time horizons contribute to the observed variations [15].

The range of schizophrenia costs and their variations among countries may result both from different health care systems and treatment applied. Patients hospitalized in Kiel were treated with neuroleptics of all generations. The most commonly used atypical antipsychotic was amisulpride, followed by risperidone and olanzapine. In the study of Daltio et al. [10], the most popular atypical neuroleptic in Sao Paulo was risperidone, followed by olanzapine. In Poznań, however, the most popular drug was olanzapine, followed by aripiprazole, while in Lviv it was clozapine, followed by risperidone [11]. Although the group of atypical neuroleptics used in these centers seems to be quite common, the demonstrated results reveal differences in schizophrenia treatment regimens which could contribute to the dissimilarity of costs of inpatient schizophrenia care.

The most popular typical antipsychotic in Kiel was haloperidol. In Poznan, Lviv [11], and Sao Paulo [10], haloperidol was likewise the leading typical antipsychotic. The ongoing and unchanged importance of haloperidol was confirmed by the study conducted by Garcia-Ruiz et al. [3]. Although in the CATIE study [16] haloperidol was excluded from the analysis and referred to as not a popular antipsychotic at the moment, haloperidol is a medicine still frequently used.

Patients hospitalized in Kiel were receiving additional pharmacotherapy as well. It is justified because adjunctive pharmacotherapy is very common in schizophrenia and leads to higher efficacy of treatment [17]. Moreover, it ensures complete treatment which is not so obvious in low- or middle-income countries [11].

Although in some countries hospitalization accounts for more than 90 % of total direct health care costs [5], it seems that comprehensive and innovative treatment may lead to significant savings of costs associated with schizophrenia. Investment in innovative pharmacotherapy will lead to increase in total cost of disorder at first [18], but will pay off in significant savings considered in the long-run [3, 5]. Furthermore, studies conducted by Peng et al. [19], Asseburg et al. [20], and Kane et al. [21] confirmed that initiation of depot antipsychotic therapy contributed to a decline in hospitalization rates and reduction of costs as well.

Depot neuroleptics were administered to some of the patients treated in Kiel. Taking into consideration efficacy of depot neuroleptics it seems that such treatment should be considered as a cost-effective investment. Nevertheless, antipsychotic treatment ought to be individually tailored to provide optimum treatment and recovery as well [22]. Therefore, there is no point in paying more e.g. for depot antipsychotics when oral medicines are effective enough. In that case, it would be better to provide several non-pharmacological therapies which are known to be an important and effective part of comprehensive treatment of schizophrenia.

In Kiel, patients affected with schizophrenia were provided not only with a suitable pharmacotherapy but also with comprehensive non-pharmacological treatment which was structured and applied to all the patients treated in Kiel. The importance of non-pharmacological therapy was confirmed by Rummel-Kluge et al. [23] who indicated that psychoeducation contributes to a better compliance, helps cope better with the mental disorder and, consequently, decreases the costs owing to less frequent and shorter inpatient stays. Moreover, social competence training addresses social functioning and helps patients have both an easier and a more effective come-back to the society during the remission stage [2]. What is also important, non-pharmacological therapies are expected by the patients [24] and help the patients get and retain a job during schizophrenia relapse. Furthermore, work is often a goal for people with schizophrenia [25], is being considered therapeutic for them, and can lead to a better quality of life, fewer hospitalizations, and, finally, to savings of direct and indirect costs of schizophrenia [26, 27].

In Kiel, there were several facilities providing the possibility of supported employment for people after mental crises. Some of them were located close to the hospital, offering both the patients and communities the opportunity to be familiarized with the course of disorder, opportunity of relieving the social stigma of schizophrenia, and of showing that people in a remission stage are capable of working and living normally in the community. The scope and the level of development of non-pharmacological therapies in Germany may account for the results obtained by Marwaha et al. [25] where the authors indicated that the employment rate of people with schizophrenia was above 30 % in Germany, compared to 11.5 % in France and 12.9 % in the UK. It confirms that there is an urgent need to develop supported employment opportunities. It will contribute to more effective treatment of schizophrenia and to significant savings both in the group of direct and indirect costs [28].

Apart from that, the appraisal of frequency of schizophrenia relapses during the last 10 years of the time of analysis provides interesting data and confirms the efficacy of very comprehensive treatment. Patients in Kiel were hospitalized less often than those in Poznań or Lviv [11]. The difference between Kiel and Lviv was significant [11]. In a group above seven inpatient stays within the last 10 years, there were 3 patients in Kiel, 16 patients in Poznań, and 27 patients in Lviv [11].

Comparing Kiel to Poznań [11], it seems that treatment regimens are similar, but in Kiel opportunities of supported employment are more popular. A significant difference is being observed especially between Kiel and Lviv, though [11]. It may stem from different health care systems, differences in the economic status, and from different political situation in these countries likewise. Contrary to Germany and Poland, there is no medicine reimbursement in outpatient care in Ukraine and patients are frequently obliged to be treated only with older neuroleptics. Therefore, atypical neuroleptics in particular are not economically available for patients treated in the Ukraine [11].

Drug reimbursement in Germany provides also access to innovative medicines. For example, original olanzapine (5 mg; 28 pills) costs EUR 5.00, and risperidone depot (37.5 mg) EUR 10.00 after reimbursement, but chronically ill patients can obtain these medicines for free. Taking into consideration the level of salaries in Germany, these prices seem nominal. Economic availability of neuroleptics also in outpatient care may contribute to a better compliance and, in association with complex non-pharmacological support, may be the main reason for lower hospitalization rates in Kiel compared to Lviv [11]. Although investment in comprehensive therapy could increase total cost of schizophrenia treatment at present, it will be cost-effective in the long term.

The present study also has some limitations. The sample of patients included in the study could be larger and individual components of hospitalization cost in Kiel could be presented as well. The system of financing of inpatient care in Kiel, however, was comprehensive and every component was covered by the value of procedure per day. Although it is difficult to compare results concerning costs of schizophrenia obtained in different countries, this study is an interesting source of data concerning important facets of schizophrenia treatment and about the economic burden of schizophrenia. Conducting analyses at academic hospitals will allow application of this unique and useful knowledge by health care decision-makers, clinicians, and students taught at academic centers.
